# Drug treatment of breast cancer brain metastases: progress and challenges

**DOI:** 10.1007/s12672-025-02820-9

**Published:** 2025-06-07

**Authors:** Jialing Luo, Aixiong Ren, Dikun Si, Jixin Yang, Dongdong Xu, Nanlin Li

**Affiliations:** Department of Thyroid, Breast and Vascular Surgery, The First Affiliated Hospital of Air Force Medical University, Xi’an, Shaanxi Province China

**Keywords:** Breast neoplasms, Brain metastasis, Antineoplastic protocols, Drug therapy

## Abstract

Breast cancer is one of the most prevalent malignancies among women worldwide, with brain metastasis occurring in approximately 10–16% of cases, significantly contributing to poor prognosis and reduced quality of life. The treatment of breast cancer brain metastasis requires a multidisciplinary approach, prioritizing local therapies such as surgery and radiotherapy to address central nervous system lesions. However, local treatments often struggle to effectively control the progression of brain metastases and are associated with multiple complications, necessitating adjunctive systemic and supportive therapies. Following local therapy, breast cancer brain metastasis patients may benefit from systemic treatments. Pharmacological therapies, including chemotherapy, targeted therapy, immunotherapy, and antibody–drug conjugates, have emerged as vital strategies for breast cancer brain metastasis treatment. Targeted therapies combined with stereotactic radiosurgery and surgical resection have shown improved survival rates. However, challenges remain, such as high costs, limited availability of radiotherapy equipment, and individualized treatment requirements based on lesion characteristics and systemic disease control. Further advancements in pharmacological options, particularly targeted and immune therapies, offer promising avenues for improving outcomes and survival in breast cancer brain metastasis patients.

## Background

Breast cancer remains one of the most prevalent malignancies among women worldwide. According to the latest data from the National Cancer Center, based on tumor registry and follow-up surveillance statistics, there were 2.3 million new cases of breast cancer globally in 2022, ranking second in incidence among malignancies, following lung cancer [1].While advancements in screening technologies and treatment approaches have improved overall survival (OS) rates, patients still face a substantial risk of distant metastasis, particularly brain metastasis (BM), which occurs in approximately 10–16% of cases and ranks second among cancers causing BM [2]. Breast cancer brain metastasis (BCBM) significantly contributes to poor prognosis, severely impairing both quality of life and OS [3].

Its management necessitates a multidisciplinary approach aimed at maximizing survival duration while striving to improve the quality of life. The treatment strategy prioritizes localized interventions, such as surgery and/or radiotherapy for the central nervous system, following a comprehensive evaluation of the patient’s systemic status to ensure stability and treatment tolerance. Despite their importance, local therapies alone often fail to control the progression of BM due to complications and are therefore complemented by systemic and supportive treatments. Clinically, BCBM management remains challenging. The blood–brain barrier (BBB) impedes the penetration of systemic therapies, limiting their effectiveness [4]. Additionally, the heterogeneity of BCBM requires individualized treatment plans, considering factors such as lesion number, size, location, and systemic disease control [5]. Consequently, while local therapies are vital for addressing isolated lesions, they are insufficient to manage diffuse or multifocal BM comprehensively [6].

In recent years, pharmacological therapy has emerged as a pivotal strategy in the treatment of BCBM, particularly with advancements in targeted therapy and immunotherapy offering renewed therapeutic prospects for patients. Pharmacological approaches encompass traditional chemotherapy, targeted therapy, and innovative treatments such as immune checkpoint inhibitors (CPIs) and antibody–drug conjugates (ADCs). In the era of targeted therapy (TT), median survival rates for BCBM patients have improved significantly through the integration of local treatments, such as stereotactic radiosurgery (SRS) and surgical resection. However, SRS is constrained by challenges including limited access to radiotherapy equipment, high treatment costs, and the need to account for the patient’s physical condition and the characteristics of brain lesions. Surgical resection, while effective in swiftly alleviating tumor-induced neurological symptoms, enhancing quality of life, and sometimes reducing local recurrence when combined with SRS, is restricted by factors such as lesion location, the patient’s overall health, and the surgical expertise available at medical institutions. Additionally, local treatments may result in serious complications, such as neurological dysfunction and cognitive impairments. Given the constraints of local treatments, extensive research into advancements in pharmacological therapies is of considerable clinical significance for improving prognosis and survival outcomes in BCBM patients. The following review examines domestic and international progress in pharmacological treatments for BCBM, aiming to inform and inspire clinical practice.

## Current status and challenges of drug therapy

**Fig. 1:**
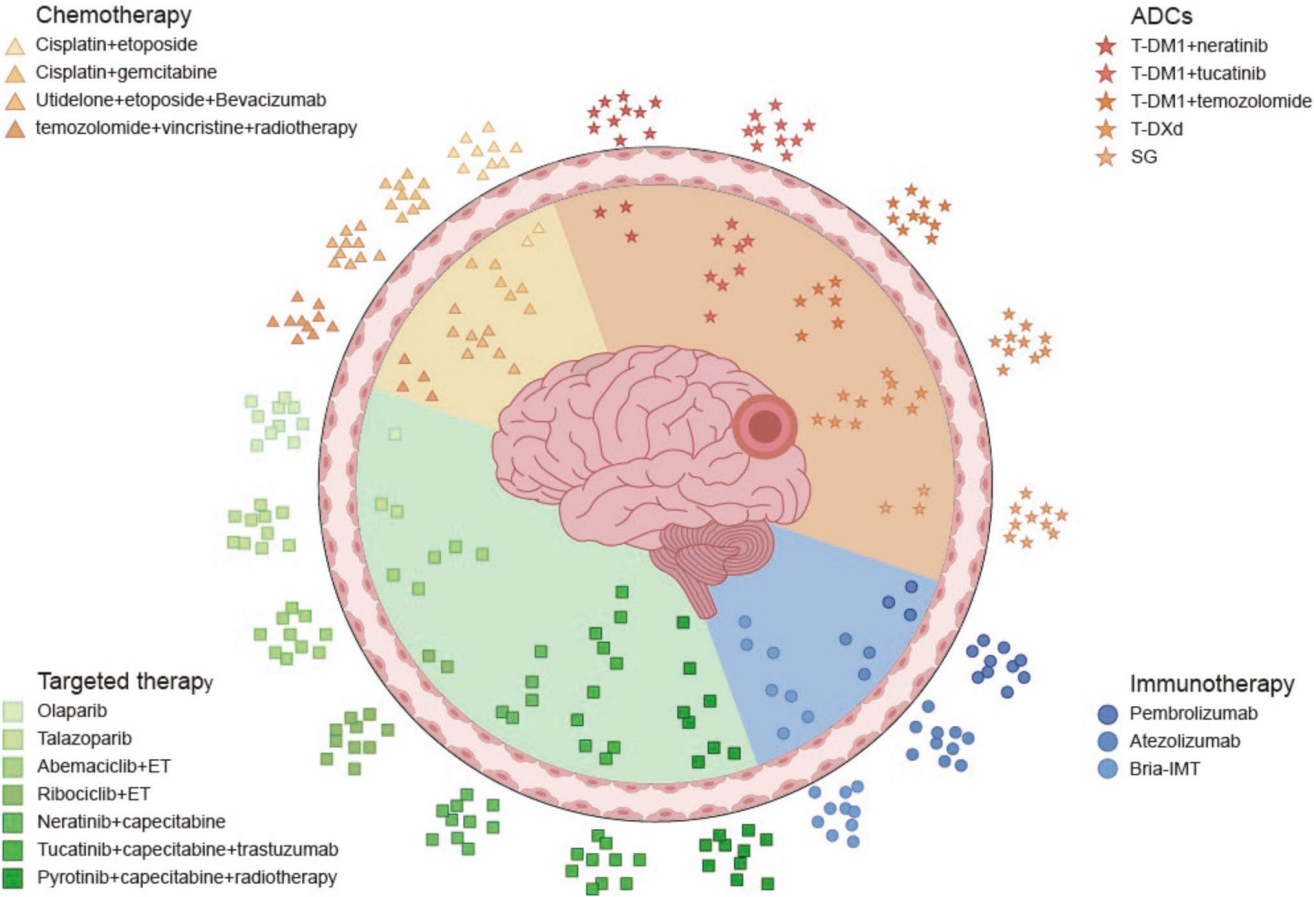
Combination of systemic therapies for brain metastasis in breast cancer

At present, there are no standardized systemic treatment regimens recommended in existing guidelines for BCBM. Recognizing that appropriate systemic therapy is an integral component of comprehensive treatment, this review introduces pharmacological options for systemic therapy, tailored to the molecular subtypes of advanced breast cancer, based on current evidence. (Fig. 1).

## HR + /HER2–BCBM

Currently, endocrine therapy (ET) combined with targeted therapy or endocrine-based treatment remains the preferred approach for HR + /HER2–metastatic breast cancer (MBC) [7]. The combination of endocrine therapy with targeted agents, including CDK4/6 inhibitors (CDK4/6i), PI3Kα inhibitors, AKT inhibitors, mTOR inhibitors, and HDAC inhibitors, particularly CDK4/6i, has emerged as the standard first- and second-line treatment for HR + /HER2–MBC. This preference is driven by its superior efficacy over endocrine monotherapy, its comparable or even greater effectiveness relative to chemotherapy, and its favorable tolerability profile [8].

### Endocrine therapy + CDK4/6i

Currently, three CDK4/6i have been approved by the U.S. Food and Drug Administration (FDA): palbociclib, ribociclib, and abemaciclib. These CDK4/6i drugs have also been approved for clinical application in China by the National Medical Products Administration (NMPA). Additionally, China has independently developed its first CDK4/6 inhibitor, dalpiciclib, which is now available for clinical adoption  domestically.

#### Palbociclib

Palbociclib demonstrated significant efficacy in improving progression-free survival (PFS) in the PALOMA-1 and PALOMA-2 clinical trials. In PALOMA-2, the combination of palbociclib and letrozole versus placebo and letrozole achieved a median PFS (mPFS) of 24.8 months compared to 14.5 months, representing a 42% reduction in risk (HR = 0.58; 95% CI 0.46–0.72; P < 0.001). Updated results from a 38-month follow-up further reinforced the superior efficacy of palbociclib, extending the mPFS to 27.6 months. Palbociclib became the first CDK4/6 inhibitor approved for the treatment of HR + /HER2–MBC, with or without visceral metastases [9, 10]. Subgroup analyses from both PALOMA-1 and PALOMA-2 demonstrated that palbociclib provided substantial benefits to patients, irrespective of the presence or absence of visceral or bone metastases [11, 12]. Although both trials permitted the inclusion of patients with BM, the number of such cases was minimal—2 in PALOMA-1 and 5 in PALOMA-2. Consequently, no subgroup analysis was performed to evaluate the specific benefits for patients with BM.

#### Ribociclib

The approval of ribociclib was driven by the success of the MONALEESA series of clinical trials, with its efficacy confirmed in the phase III MONALEESA-2, MONALEESA-3, and MONALEESA-7 studies. In first-line treatment for advanced breast cancer, ribociclib combined with endocrine therapy demonstrated significant improvements in PFS for both postmenopausal women (MONALEESA-2) and premenopausal women (MONALEESA-7). In MONALEESA-2, a second interim analysis with a median follow-up of 26.4 months reported a mPFS of 25.3 months for ribociclib plus letrozole compared to 16.0 months for placebo plus letrozole, reflecting a 43% risk reduction (HR = 0.57; 95% CI 0.457–0.704) [13]. Similarly, in MONALEESA-7, the ribociclib group achieved a mPFS of 23.8 months versus 13.0 months for the placebo group, corresponding to a 45% risk reduction (HR = 0.55; 95% CI 0.44–0.69; P < 0.0001) [14]. For postmenopausal women who had previously received endocrine therapy, ribociclib combined with fulvestrant in the MONALEESA-3 trial demonstrated significant benefits in both PFS and OS compared to placebo plus fulvestrant. The mPFS was significantly prolonged to 20.5 months in the ribociclib group versus 12.8 months in the placebo group (HR = 0.593; 95% CI 0.480–0.732; P < 0.001) [15]; At a median follow-up of 70.8 months, the median OS (mOS) reached 67.6 months for ribociclib compared to 51.8 months for placebo (HR = 0.67; 95% CI 0.50–0.90) [16]. Across all MONALEESA trials, ribociclib maintained health-related quality of life without adverse impacts. However, patients with BM were excluded from MONALEESA-2 and MONALEESA-7. In MONALEESA-3, among the 726 patients randomized (2:1) to receive ribociclib plus fulvestrant or placebo plus fulvestrant, 8 patients presented with stable BM. Nevertheless, specific central nervous system outcomes for these patients were not reported.

#### Abemaciclib

Abemaciclib received FDA approval for its ability to significantly enhance the efficacy of endocrine therapy in both first-line and second-line settings. In the MONARCH-3 international clinical trial, conducted in endocrine-sensitive or untreated patients, the combination of abemaciclib and an aromatase inhibitor (AI) was compared to placebo and AI. The results demonstrated a significantly higher tumor response rate (49.7 vs. 37%) and nearly doubled PFS at 28.2 months versus 14.8 months (HR = 0.540; 95% CI 0.418–0.698) [17]. The efficacy of abemaciclib was further validated in the MONARCH-2 large-scale controlled trial, which included postmenopausal women with HR + /HER2–MBC who had progressed during prior ET. The combination of abemaciclib and fulvestrant achieved a mPFS of 16.9 months compared to 9.3 months in the control group, along with a mOS of 46.7 months versus 37.3 months [18]. To assess the efficacy of abemaciclib in the Chinese population, the MONARCH-Plus trial, a multinational, randomized phase III study, primarily focused on Chinese patients. The findings indicated that abemaciclib combination therapy provided benefits across various subgroups, regardless of endocrine resistance, liver metastasis, or age over 65 years [19]. Notably, these clinical trials excluded patients with BM. However, abemaciclib, approved for the treatment of HR + /HER2–MBC, has demonstrated the ability to cross the BBB, achieving therapeutic concentrations in cerebrospinal fluid (CSF) comparable to those in plasma. [20]. A phase II clinical trial subsequently evaluated the activity and safety of abemaciclib monotherapy versus combination with ET in HR + MBC patients with secondary BM. In cohort A (HR + /HER2–MBC, n = 58), three patients were identified as responders, resulting in an intracranial objective response rate (iORR) of 5.2% (95% CI 0.0–10.9%). Although the study did not meet the primary endpoint of achieving an iORR ≥ 15%, the data suggested that abemaciclib provides clinical benefits for HR + /HER2–MBC patients. In heavily pretreated HR + /HER2–MBC patients, the intracranial clinical benefit rate (iCBR) was 24% (95% CI 13.1–35.2%), with a mOS of 12.5 months (95% CI 9.3–16.4). Pharmacokinetic analyses revealed that abemaciclib and its active metabolites achieved concentrations in BM tissues far exceeding the levels required for CDK4/6 inhibition [21]. Given the therapeutic challenges in MBC patients with central nervous system (CNS) involvement, these findings underscore the need for further investigations, including the evaluation of novel abemaciclib-based combination therapies.

#### Dalpiciclib

Dalpiciclib, the first domestically developed, highly selective CDK4/6i in China, was designed to address the specific clinical needs and characteristics of the Chinese population. The DAWNA-1 trial, a phase III randomized controlled study, evaluated HR + MBC patients who had progressed on prior ET. This trial demonstrated that the combination of dalpiciclib and fulvestrant significantly improved PFS to 15.7 months compared to 7.2 months with fulvestrant monotherapy, representing an extension of 8.5 months and a 58% reduction in the risk of disease progression (HR = 0.42; 95% CI 0.31–0.58). These results underscore the efficacy of dalpiciclib combined with fulvestrant as a second-line treatment for HR + MBC [22]. The DAWNA-2 study further evaluated the efficacy and safety of dalpiciclib in combination with letrozole or anastrozole as a first-line treatment for HR + /HER2–MBC. The results revealed a significant extension in PFS, reaching 30.6 months versus 18.2 months, corresponding to a 12.4-month improvement and a 49% reduction in the risk of disease progression (HR = 0.51; 95% CI 0.38–0.69) [23]. The DAWNA-2 study established new benchmarks for PFS and overall response rate (ORR, complete response + partial response) in the first-line treatment of HR + /HER2- MBC using CDK4/6i combined with non-steroidal a AIs, providing robust evidence for the clinical application of dalpiciclib. However, this study excluded patients with BM. In the DAWNA-1 trial, exclusion criteria included leptomeningeal metastases or untreated CNS metastases. Patients with previously treated BM—who were clinically stable for at least one month on imaging, off systemic corticosteroid therapy (≥ 10 mg/day prednisone or its equivalent for more than two weeks), and asymptomatic—were eligible if they had undergone local treatments such as radiotherapy or surgery. Unfortunately, no analyzable data were available for this subgroup, leaving the efficacy of dalpiciclib in patients with BM unresolved.

Studies have identified that factors such as positive lymph node status, high histological grade, younger age, high tumor burden, and the presence of BRCA1/2 gene mutations are considered high-risk factors for the development of BM [24, 25]. A key question to explore is whether the early use of CDK4/6i can help prevent CNS metastases. The PENELOPE-B study investigated HR + /HER2- early breast cancer patients with residual invasive disease following neoadjuvant chemotherapy and surgery, who were at high risk of recurrence. It assessed the efficacy and safety of palbociclib combined with standard ET versus placebo plus ET. After a median follow-up of 42.8 months, the 3-year invasive disease-free survival (iDFS) rate was 81.2% in the palbociclib group compared to 77.7% in the placebo group (HR = 0.93; P = 0.525), failing to achieve a statistically significant benefit [26]. The NATALEE study evaluated the addition of 3 years of ribociclib to standard adjuvant ET in stage II and III breast cancer, including lymph node-negative patients (T2N0 with histological grade 3, grade 2 with Ki-67 > 20%, or high-risk genetic profiles). Preliminary results demonstrated a 25.1% reduction in recurrence risk, with 3-year iDFS rates of 90.7% versus 87.6% (HR = 0.749; P = 0.0012), although this indication has yet to receive approval [27]. The MONARCH-E study enrolled HR + /HER2- breast cancer patients with ≥ 4 positive lymph nodes or 1–3 positive nodes accompanied by high-risk features, such as histological grade 3, tumor size > 5 cm, or Ki-67 > 20%. Adding 2 years of abemaciclib to ET following (neo)adjuvant chemotherapy significantly reduced the risk of recurrence, yielding absolute iDFS benefits of 3.5% at 2 years [28] and 6.4% at 4 years [29]**;** The latest findings reveal sustained benefit, with an absolute iDFS improvement of 7.6% and a 32% relative risk reduction in iDFS events compared to standard ET alone (83.6% vs. 76.6%; HR = 0.680; P < 0.001) [30]. For HR + /HER2- breast cancer patients with ≥ 4 positive lymph nodes, adding 2 years of abemaciclib to standard adjuvant ET is strongly recommended; for patients with 1–3 positive lymph nodes and high-risk features (e.g., histological grade 3 or tumor size > 5 cm), 2 years of abemaciclib is also advised. Some researchers propose extending this recommendation to patients with 1–3 positive nodes, no grade 3 or T3 disease, but a Ki-67 proliferative index ≥ 20%. Although CNS events at first recurrence are uncommon in HR + /HER2–MBC, extended follow-up may determine whether adjuvant abemaciclib could help delay or prevent the development of BM. These findings highlight the potential role of CDK4/6i in reducing the risk of metastasis to critical sites, such as the CNS, underscoring their importance in optimizing long-term outcomes for high-risk patients.

### Chemotherapy

Current treatment guidelines for HR + /HER2–MBC recommend sequential endocrine and targeted therapy until available options are exhausted, followed by systemic cytotoxic chemotherapy. However, the applicability of this approach to BCBM remains uncertain. Although cytotoxic agents may offer a more rapid response compared to some targeted or endocrine therapies in the context of BCBM, their use is often accompanied by significant toxicity, as discussed in the chemotherapy section of the TNBCBM chapter.

### Discussion

Based on the presented data, combination therapy with ET and CDK4/6i, or endocrine-based regimens, is unequivocally the preferred strategy for HR + /HER2–MBC. However, the efficacy of CDK4/6i in HR + /HER2–BCBM remains an open question. A phase II clinical study evaluating the iORR of abemaciclib in HR + BCBM patients did not meet its primary endpoint. Nevertheless, the study confirmed that abemaciclib achieves therapeutic concentrations in the CNS and exhibits activity in BCBM. SPH4336, a novel, highly selective oral CDK4/6i, has demonstrated significant potential in breast cancer treatment (Clinical Trial Registration Number: NCT05905614) [31]**,** A phase II clinical study (CTR20231425) is currently ongoing to evaluate its combination with ET in HR + /HER2–MBC with BM, offering renewed hope for BCBM patients. Looking ahead, the development and emergence of more effective CDK4/6i hold promise for advancing treatment options and improving outcomes in HR + BCBM.

## HER2 + BCBM

Recent advancements in the pharmacological interventions of BCBM has focused predominantly on HER2-positive breast cancer (HER2+ BC). Significant advancements in mechanistic research and systemic therapies have challenged the traditional belief that large-molecule drugs are ineffective in treating HER2 + BCBM due to their limited ability to cross the BBB. Systemic treatment options for HER2 + BCBM now encompass not only small-molecule HER2-targeting tyrosine kinase inhibitors (TKIs) but also large-molecule therapies such as monoclonal antibody and ADCs, marking a significant breakthrough in treatment strategies (Table 1).

**Table 1:** Selection of relevant studies evaluating systemic therapy options for HER2 + BCBM

Trial ID and/or name	Regimen	Trial description	Outcomes for BMs
LANDSCAPE (NCT00967031)	Lapatinib + capecitabine	HER2-positive MBC pts, pre-treated with TTZ, capecitabine and lapatinib-naïve and without indication for surgical resection of BMs	CNS-ORR: 65.9%
TBCRC 022 (NCT01494662)	Neratinib + capecitabine	HER2 + MBC with measurable,progressive BMs (lapatinib-naïve and lapatinib-treated cohorts)	CNS-ORR:49%; mPFS: 5.5mo (lapatinib-naïve cohorts) CNS-ORR:33%;mPFS:3.1mo (lapatinib-treated cohorts)
NALA (NCT01808573)	Neratinib + capecitabine	HER2 + MBC ≥ lines of systemic treatment, including pts with asymptomatic or stable(treated or untreated) BMs	CNS-ORR:26.3%
HER2CLIMB (NCT02614794)	Tucatinib + trastuzumab + capecitabine	HER2-positive mBC, pre-treated with trastuzumab, pertuzumab, and T-DM1, progressing or stable, including untreated, BMs	CNS-mPFS:13.9mo(stable BMs) CNS-mPFS:9.6mo(avtive BMs)
PERMEATE (NCT03691051)	Pyrotinib + capecitabine	HER2 + BCBM RT-naive BMs(cohort A) and RT-treated BMs (cohort B)	**cohort A: **CNS-ORR:74.6% mPFS:11.3mo **cohort B: **CNS-ORR:42.1% mPFS:5.6mo
NA (NCT04582968)	Pyrotinib + capecitabine + radiotherapy	HER2 + BCBM	CNS-ORR:85% mPFS:17.6mo
TBCRC022's cohort 4 (NCT01494662)	Neratinib + T-DM1	HER2 + MBC with measurable,progressive BMs (cohort 4A-previously untreated BCBM, cohorts 4B and 4C-BCBM progressing after local CNS-directed therapy without (4B) and with (4C) prior exposure to T-DM1)	CNS-ORR:33.3%(cohort 4A) CNS-ORR:35.3%(cohort 4B) CNS-ORR:28.6%(cohort 4C)
DESTINY-Breast01/-02/-03	trastuzumab deruxtecan (T-DXd)	This exploratory pooled analysis investigated the efficacy and safety of trastuzumab deruxtecan (T-DXd) versus comparator treatment in patients with HER2 + MBC BMs at baseline, categorized according to previous local treatment	**treated/stable BMs: **CNS-ORR:45.2% mCNS-PFS:12.3mo **untreated/active BMs: **CNS-ORR:45.5% mCNS-PFS: 18.5mo
DEBBRAH NCT04420598	trastuzumab deruxtecan (T-DXd)	DEBBRAH is a single-arm, five-cohort, phase II study evaluating T-DXd in patients with central nervous system involvement from HER2-positive and HER2-low MBC. Here are results from patients with non-progressing BMs after local therapy (n = 8; cohort 1), asymptomatic untreated BMs (n = 4; cohort 2), or progressing BMs after local therapy (n = 9; cohort 3)	**cohort 1: **16 week PFS rate:87.5% ORR:66.7% **cohort 2: **iORR:50% ORR:50% **cohort 3: **iORR:44.4% ORR:66.7%
TUXEDO-1 (NCT04752059)	trastuzumab deruxtecan (T-DXd)	HER2 + BC pts with newly diagnosed or progressing BMs after prior local therapy; and/or prior exposure to dual blockade and without indication for immediate local therapy	iRR:73.3% mPFS:21mo
DESTINY-Breast12 (NCT04739761)	trastuzumab deruxtecan (T-DXd)	HER2^+^ MBC from two separate cohorts of patients with and without baseline BMs. Patients with HER2 + MBC with previously treated and stable or active (untreated or previously treated and progressing) BMs	BMs cohort: 12 month PFS rate: 61.6% mPFS:17.3%

ID: identifier; iRR: intracranial response rate; iORR: intracranial objective response rate; BMs: brain metastasis; BCBM: breast cancer with brain metastasis; BC: breast cancer; CNS-ORR: central nervous system objective response rate; MBC: metastasis breast cancer; mPFS: median progression-free survival; mo: months

### TKIS

The four approved small-molecule TKIs have shown promising efficacy in treating HER2 + BCBM.

#### Lapatinib

Lapatinib, a small-molecule TKI that dual-targets EGFR and HER2 [32]. Has demonstrated clinical benefit in HER2+ MBC patients with untreated BM in the phase II LANDSCAPE trial, the combination of lapatinib and capecitabine achieved a CNS objective response rate (CNS-ORR) of 65.9%, a CNS progression-free survival (CNS-PFS) of 5.5 months, and a median time to radiotherapy of 8.3 months [33]. However, a meta-analysis of 12 studies involving HER2+ MBC patients with BM reported a CNS-ORR of only 29.2% for lapatinib plus capecitabine, suggesting slightly lower efficacy compared to tucatinib-based regimens [34].

#### Neratinib

Neratinib, an irreversible HER2 inhibitor targeting HER1/HER2/HER4, has been approved for the adjuvant treatment of early-stage HER2+ breast cancer [35, 36]. Its ability to penetrate the BBB positions it as a promising therapeutic option for BM. In the phase II NEfERT-T trial, which included patients with stable BM, the combination of neratinib and paclitaxel demonstrated a lower CNS recurrence rate compared to trastuzumab plus paclitaxel (8.3% vs. 17.3%; RR = 0.48, 95% CI 0.29–0.79; P = 0.002) and delayed CNS progression (HR = 0.45; 95% CI 0.26–0.78; P = 0.004). However, these findings require further validation in larger studies [37, 37]. In the phase II TBCRC 022 trial, neratinib plus capecitabine was assessed in TKI-naive HER2+ breast cancer patients with CNS metastases. In cohort 3A, the CNS-ORR was 49%, with a mPFS of 5.5 months and a mOS of 13.3 months. In cohort 3B, which included patients previously treated with lapatinib (with slow enrollment), the CNS-ORR was 33%, mPFS was 3.1 months, and mOS was 15.5 months, achieving the study's expected endpoints [38]. The phase III NALA trial evaluated HER2+ MBC patients who had received ≥ 2 lines of prior anti-HER2 therapies, randomizing them to neratinib plus capecitabine or lapatinib plus capecitabine. The neratinib group demonstrated intracranial activity, with a median duration of response(DoR) of 8.5 versus 5.6 months (*P* = 0.0004) and fewer patients required interventions for symptomatic CNS metastases (22.8% vs. 29.2%; P = 0.043) [39]. These results highlight neratinib’s potential to delay CNS progression and provide intracranial benefit in HER2+ breast cancer patients with BM, particularly when combined with capecitabine. Further studies are warranted to confirm its efficacy in this population.

#### Tucatinib

The HER2CLIMB study demonstrated significant efficacy of the combination of tucatinib, trastuzumab, and capecitabine (XH) in patients with locally advanced, unresectable, or metastatic HER2+ BC, including those with BM [40]. Among the study population, 47% (198 patients) had baseline BM, including 37 with stable and 56 with active BM. Subgroup analysis revealed that, at a median follow-up of 29.6 months, the tucatinib group achieved a significantly longer mOS compared to the placebo group, with an OS difference of 9.1 months (21.6 months vs. 12.5 months). In patients with active BM, the OS was 21.4 months compared to 11.8 months, while in those with stable BM, the OS was 21.6 months versus 16.4 months. The mPFS was also notably improved in the tucatinib group, reaching 9.9 months compared to 4.2 months in the placebo group. In terms of intracranial responses, the CNS-PFS showed significant differences: 9.6 months versus 4.0 months in patients with active BM and 13.9 months versus 5.6 months in those with stable BM. These findings align with the overall benefit observed in patients with BM, furthermore, the CNS-ORR was 47.3% in the tucatinib group, significantly higher than the 20.0% observed in the placebo group, with a medianDoR of 8.6 months versus 3.0 months[41]. The HER2CLIMB study provides the first conclusive evidence of an OS benefit in HER2+ BC patients with BM, addressing a critical unmet medical need in this population, where CNS metastases occur in up to 50% of patients[42]. These results have led to the ESMO/ABC guidelines recommending tucatinib as the first-line treatment for patients with active HER2+ BCBM.

#### Pyrotinib

Pyrotinib, an oral small-molecule TKI targeting HER1, HER2, and HER4, has shown promising efficacy in HER2+ BCBM [43]. In the PERMEATE trial, the combination of pyrotinib and capecitabine demonstrated substantial ORR and significantly prolonged PFS in HER2+ BCBM patients. For patients without prior local radiotherapy, the combination achieved a mPFS of 11.3 months and a CNS-ORR of 74.6%, comparable to responses seen in extracranial lesions. In patients with BM that had progressed following local radiotherapy, the mPFS was 5.6 months, with a CNS-ORR of 42.1% [44]. Some studies suggest that pyrotinib may enhance the radiosensitivity of HER2+ BM [45], prompting exploration into whether adding radiotherapy could further improve CNS-PFS when combined with pyrotinib and capecitabine. A single-center, single-arm, non-randomized Phase Ib/II clinical trial investigated this triple combination. With a median follow-up of 13.7 months, the results showed a CNS-PFS of 18.0 months (95% CI 15.5–NR), a CNS-ORR of 85% (34/40), and a CNS-PFS rate of 74.9% (95% CI 61.9–90.7%), surpassing the pre-specified endpoint of 70%. The most common grade 3/4 treatment-related adverse event was diarrhea (3/40) [46]. These findings suggest that the combination of radiotherapy, pyrotinib, and capecitabine offers durable intracranial benefit for HER2+ BCBM patients and underscores the need for large-scale randomized Phase III clinical trials.

Collectively, these studies highlight the efficacy of combination therapies involving small-molecule TKIs in treating BM, likely due to the ability of small molecules to penetrate the BBB more effectively. Although no head-to-head comparisons exist, cross-study analyses suggest that the combination of tucatinib, trastuzumab, and capecitabine stands out with particularly promising results. This regimen has been recognized in major guidelines as a leading treatment option for HER2+ BC patients with BM. Looking forward, the integration of radiotherapy with TKIs and capecitabine holds potential for even greater therapeutic benefit. This innovative approach offers renewed hope for improving outcomes and expanding treatment options for this challenging patient population.

### Monoclonal antibody

Trastuzumab is a milestone in the treatment of HER2+ BC, carrying revolutionary significance. It has changed the treatment paradigm for HER2+ BC, marking a transition from chemotherapy to precise targeted therapy. This advancement has ushered breast cancer into an era of individualized treatment guided by molecular subtyping. From adjuvant therapy, neoadjuvant therapy, to late-stage salvage treatment, trastuzumab covers the entire course of HER2+ BC. However, due to its large molecular size, its cerebrospinal fluid concentration is only 0.1–1.0% of the plasma concentration under conventional treatment methods and dosages[47]. Nevertheless, due to the outstanding efficacy of its combination with pertuzumab and chemotherapy, the "dual-target" regimen with taxane-based drugs remains the first-line treatment of choice for HER2+ MBC, regardless of the presence of BM. The CLEOPATRA study showed that the dual-target regimen combined with pertuzumab can extend the median time to BM by 3.1 months and prolong the mOS by 8.1 months [48].

Considering that the presence of the BBB may be a major factor limiting trastuzumab from reaching therapeutic concentrations in the cerebrospinal fluid, studies have explored the efficacy of intrathecal injection of trastuzumab for the treatment of leptomeningeal disease (LMD) [49–51]. However, a 2023 meta-analysis by Lazaratos A.M and colleagues showed that compared to oral or intravenous HER2-targeted therapies, intrathecal injection of trastuzumab did not significantly improve OS or CNS-PFS in patients with LMD [52].

The CLEOPATRA study has already confirmed that systemic injection of trastuzumab plus pertuzumab is significantly superior to trastuzumab alone. But is intrathecal injection of the “dual-target” regimen safe and effective for patients with LMD? A single-center, non-randomized, phase I clinical study, published online on May 23, 2024, in JAMA Oncology, showed that intrathecal injection of trastuzumab and pertuzumab after radiotherapy was well-tolerated in HER2+ LMD patients (with recommended doses of 80 mg for trastuzumab and 80 mg for pertuzumab). Finally, intracranial responses appeared encouraging: 2 patients (2/9) achieved complete remission, 3 patients (3/9) had partial remission, and 4 patients (4/9) had stable disease; moreover, the observed OS was higher than that of historical control participants [53]. Given the favorable safety profile observed in this phase I study, phase II is currently ongoing, enrolling patients to assess OS.

### ADCS

It has long been assumed that large-molecule drugs face challenges in crossing the BBB, making small-molecule agents more suitable for the treatment of BCBM. However, recent advancements have positioned ADCs as a promising new class of therapeutics. ADCs are an innovative form of targeted therapy that harnesses monoclonal antibodies to deliver highly potent cytotoxic agents directly to cancer cells. By linking a cytotoxic payload to a tumor-specific antibody via a specialized linker, ADCs enhance the efficacy of chemotherapy while minimizing systemic toxicity. Notably, ADCs exhibit a bystander effect, wherein the cytotoxic payload can diffuse to adjacent tumor cells, including those lacking target antigen expression, thereby exerting cytotoxic effects on nearby cancer cells. This novel drug design has sparked considerable interest among researchers, leading to investigations into the potential role of ADCs in addressing the challenges of BM in breast cancer.

#### T-DM1

T-DM1, the first approved ADC in China, is widely used for the treatment of breast cancer. It comprises trastuzumab (T) conjugated to the chemotherapy agent emtansine (DM1) via a stable thioether linker.

As a monotherapy, T-DM1 has demonstrated efficacy in small to medium-sized clinical trials for HER2+ BCBM. A retrospective study from France (BCRT) analyzed 39 patients with BM 36 of whom had undergone prior local treatment. The mPFS was 6.1 months, while mOS had not yet been reached [54]. Similarly, the KAMILLA study showed that T-DM1 could delay BM progression in HER2+ MBC patients previously treated with trastuzumab and taxane-based therapy. In a subgroup analysis of 398 patients with BM, among the 126 patients with measurable brain lesions, the best ORR was 21.4% (27/126; 95% CI 14.6–29.6%) at a median follow-up of 20.6 months. The clinical benefit rate (complete response + partial response + stable disease ≥ 6 months) was 42.9%, with a median CNS-PFS of 5.5 months (95% CI 5.3–5.6) and a total OS of 18.9 months (95% CI 17.1–21.3), with no severe adverse events reported [55]. These findings suggest that T-DM1 monotherapy is a viable treatment option for HER2 + BCBM, though caution is warranted given the limitations of retrospective studies and post-hoc analyses.

Experimental models have demonstrated that combining neratinib, a HER2-targeting TKI, with T-DM1 produces a synergistic anti-cancer effect [56], preclinical evidence indicates that neratinib can overcome T-DM1 resistance and achieve more frequent and durable intracranial responses [57]. The TBCRC 022 trial (NCT01494662), which began in 2018, evaluated the CNS efficacy of neratinib-based regimens [38, 58, 59], The fourth arm of this study specifically investigated the combination of neratinib with T-DM1 in HER2 + BCBM patients [60]. Cohorts 4A, 4B, and 4C included patients with previously untreated BCBM (4A), patients who progressed after local CNS-directed therapy but had not received prior T-DM1 (4B), and those who progressed after local therapy and prior T-DM1 (4C). Cohorts enrolled 6, 17, and 21 patients, respectively. Due to slow recruitment, the study stopped after enrolling 30, 85, and 87.5% of the target participants. CNS-ORRs for cohorts 4A, 4B, and 4C were 33.3% (95% CI 4.3–77.7%), 35.3% (95% CI 14.2–61.7%), and 28.6% (95% CI 11.3–52.2%), respectively. Additionally, 38.1–50% of patients achieved stable disease or a response lasting ≥ 6 months. mOS was 30.2 months (95% CI 21.9 months–NR) in cohort 4A, 23.3 months (95% CI 17.6 months–NR) in cohort 4B, and 20.9 months (95% CI 14.9 months–NR) in cohort 4C. These results demonstrated that neratinib combined with T-DM1 provides intracranial activity, even in patients with prior T-DM1 exposure, suggesting a synergistic therapeutic effect [60]**.** Building on this synergy, researchers explored combining tucatinib with T-DM1. Preclinical data showed enhanced anti-tumor activity and synergistic cytotoxic responses, including in patient-derived xenograft (PDX) models where T-DM1 monotherapy proved ineffective. In a BM xenograft model, tucatinib penetrated intracranial tumor tissues, inhibited tumor growth, and improved survival, positioning tucatinib as a potentially optimal TKI partner for HER2-targeted therapy in HER2 + BCBM [61]**.**

Additionally, temozolomide (TMZ), an FDA-approved agent capable of crossing the BBB, has shown promise in preclinical models. Research demonstrated that administering low-dose TMZ rhythmically in a preventive manner significantly reduced BM development in murine models [62]. Building on these findings, a secondary prevention clinical trial was initiated, treating HER2 + BC patients with BM post-local therapy with oral TMZ and T-DM1 for systemic disease control. The primary endpoint was the absence of new BM within one year [63]. Results indicated that rhythmic low-dose TMZ combined with standard-dose T-DM1 exhibited potential activity in preventing local recurrence in HER2 + BCBM patients, offering a promising new approach for secondary prevention [64].

#### T-DXd

T-DXd, a HER2-targeted ADC, offers significant advancements over T-DM1, including a higher drug payload and a stronger bystander effect.

An exploratory pooled analysis from the DESTINY-Breast 01, 02, and 03 studies in patients with BM demonstrated that T-DXd significantly improved iORR compared to the control group (T-DM1 or physician's choice of therapy). The iORR was 45.2% in patients with stable BM and 45.5% in those with untreated/active BM. T-DXd also notably prolonged CNS-PFS, with mPFS of 12.3 months in patients with stable BM and 18.5 months in those with untreated/active BM. The median DoR reached 12.3 months in previously treated BM and 17.5 months in untreated/active BM, significantly reducing the risk of intracranial disease progression [65]. This analysis, the first to report T-DXd's intracranial efficacy in untreated/active BM within a randomized trial, supports the ongoing phase III DESTINY-Breast 12 trial, designed to prospectively evaluate T-DXd's efficacy in BM patients.

The DEBBRAH study explores T-DXd's role in MBC with varying HER2 expression, including stable, active, and leptomeningeal BM. Preliminary data indicate that T-DXd demonstrates efficacy in HER2 + BCBM patients, including those with untreated or active disease [66]. Notably, in patients with untreated, pathologically confirmed leptomeningeal metastases, T-DXd achieved a mOS of 13.3 months and a mPFS of 8.9 months, with a safety profile consistent with earlier findings [67]. T-DXd also showed promising efficacy in HER2-low expressing breast cancer patients with leptomeningeal metastases, without new safety concerns. These findings mark a potential paradigm shift, suggesting the possibility of transforming this difficult-to-treat condition into a more manageable disease, although further investigation is required.

The TUXEDO-1 Phase II trial assessed the activity and safety of T-DXd as systemic therapy in HER2 + MBC patients without immediate indications for local therapy. In the intention-to-treat (ITT) population, T-DXd achieved an intracranial response rate of 73.3% (11/15). After a median follow-up of 26.5 months, the mPFS was 21 months, while mOS had not yet been reached [68].

At the recent ESMO conference, the results of the DESTINY-Breast 12 trial, published in *Nature Medicine*, were unveiled. The trial included approximately 500 HER2 + MBC patients with fewer than two prior treatment lines, more than half of whom had baseline BM. In the BM cohort, the mPFS reached 17.3 months, with a 12-month PFS rate of 61.6%. The 12-month CNS-PFS rate was similarly high at 58.9%, underscoring T-DXd's comparable systemic and intracranial efficacy. In the non-BM cohort, the ORR was 62.7% and the 12-month OS rate was approximately 90% in both cohorts. Furthermore, T-DXd demonstrated durable clinical activity across the entire BM cohort, including patients with both stable and active BM. Of particular note, T-DXd exhibited an exceptional intracranial response rate in patients with active BM, with the CNS-ORR exceeding 60% in the untreated active BM subgroup. These findings highlight T-DXd as a promising first-line systemic treatment option for HER2 +BC   patients with BM, addressing a longstanding unmet need with robust and durable intracranial efficacy [69].

### Discussion

Previous clinical data have established the efficacy of TKI-based therapies in patients with BM, particularly the combination of tucatinib (T) + trastuzumab (H) + capecitabine (X). Additionally, the combination of the TKI neratinib with the novel ADC T-DM1 has shown promising results, offering a potential future treatment option for HER2 + BCBM, including the combination of tucatinib with T-DM1. While cross-trial comparisons have inherent limitations, the long-term results from the TUXEDO-1 study—demonstrating an intracranial response rate of 73.3% and a CNS-PFS of 21 months—are comparable to those from the key T-DXd trial, which reported an intracranial response rate of 44.5% and CNS-PFS of 18.5 month. Together with the favorable findings from the phase II DEBBRAH study, these results support the use of T-DXd as a second-line standard treatment for HER2 + MBC, irrespective of the presence of BM.

The latest results from the DESTINY-Breast 12 study further reinforce the efficacy of T-DXd in BM patients, achieving high ORR even with systemic treatment alone. In patients with active BM, the CNS-ORR exceeded 80%, aligning with data from the TUXEDO-1 study (CNS-ORR of 73.3%). These findings highlight the significant potential of T-DXd as a first-line treatment for patients with untreated active BM, suggesting that local therapy may not necessarily be the initial option for such patients. In clinical practice, close collaboration with radiologists and neurosurgeons is essential to determine which patients would benefit most from systemic therapy upfront and which should be prioritized for local treatments to optimize survival outcomes.

Current guidelines recommend that isolated BM lesions after progression may be considered for local treatment while continuing anti-HER2 therapy. Moreover, the potential of rhythmic low-dose TMZ in combination with standard-dose T-DM1 for secondary prevention of HER2 + BCBM following local therapy could transform current treatment strategies. In Germany, clinicians have favored tucatinib for active BM patients based on the HER2CLIMB study results. However, with the impressive CNS-ORR rate exceeding 80% in the untreated active BM subgroup from the DESTINY-Breast 12 study suggests the need to reconsider this approach. T-DXd could be introduced earlier as first-line therapy in patients with active BM, reserving tucatinib for subsequent lines upon disease progression.

Ongoing studies are exploring the potential of combining TKIs with ADCs and radiotherapy with ADCs in HER2 + BCBM. With the release of more groundbreaking data, treatment options for HER2 + BCBM will continue to expand, offering new hope for improved outcomes in this challenging clinical setting.

## TNBC BCBM

Currently, there is no established standard treatment for BM in triple-negative breast cancer (TNBC). Chemotherapy remains a cornerstone of systemic therapy for TNBC, including patients with BM, with commonly utilized agents such as capecitabine, gemcitabine, and platinum-based chemotherapeutics (e.g. carboplatin and cisplatin). However, the limited ability of conventional chemotherapy drugs to penetrate the BBB often results in suboptimal efficacy compared to targeted therapies used for HER2 + BCBM. In recent years, peptide-drug conjugates (PDCs) targeting brain metastases, such as GRN1005, as well as the development of immunotherapies and the proposal of HER2-low expression, have brought a glimmer of hope to patients with TNBCBM. Additionally, various new targeted therapies have also been actively explored in TNBC patients with BM (Table 2).

**Table 2:** Selection of relevant studies evaluating systemic therapy options for breast cancer with BM

Trial ID and/or name	Regimen	Trial description	Outcomes for BMs
NA	Cisplatin + etoposide	BCBM(previously untreated with radiotherapy)	CR:13%;PR:25%;NC:21.4%
NA	Cisplatin + Gemcitabine	Eighteen BC patients with BM who were treated with Cisplatin plus Gemcitabine regimen(15 patients (83.3%) as first-line chemotherapy, in 2 as second- line and in 1 as third-line after diagnosis of BM)	TNBC: ORR:66.6%;mPFS:7.4mo mPFS:9.2mo(regimen as first line)
UTOBIA-BM NCT05781633	Utidelone + etoposide + bevacizumab	HER2- BC pts with BM were enrolled and Simon’s two-stage optimal trial design was used for this trial (NCT05781633). If more than 3 out of 13 pts show central nervous system (CNS) response, 30 more pts would be further enrolled	CNS-ORR:73%
U-BOMB NCT05357417	Utidelone + bevacizumab	≥ 18 years old with either radiotherapy-naive or progressive BM post-radiotherapy, presented with asymptomatic or symptomatic BM associated with HER2-BC	CNS-ORR:43.5%;mPFS:7.7mo TNBC: CNS-ORR:55.0%;mPFS:8.4mo
NA	Temozolomide + vinorelbine + radiotherapy	> 18 years with histologically confirmed diagnosis of BC and newly diagnosed BMs (≥ 3), with at least one measure able lesion on brain magnetic resonance imaging (MRI)	ORR:52%; mPFS:8mo
DEBBRAH NCT04420598	T-DXd	DEBBRAH is a single-arm, five-cohort, phase II study evaluating T-DXd in patients with central nervous system involvement from HER2-positive and HER2-low MBC. Here are results from patients with heavily pretreated HER2-low MBC and active BMs, enrolled in cohorts 2 (n = 6, asymptomatic untreated BMs) and 4 (n = 6, progressing BMs after local therapy)	mPFS:5.4mo cohorts 2: CNS-ORR: 50.0% cohorts 4: CNS-ORR: 33.3%
NA	Bria-IMT^™^	Breast cancer metastasis (spread of the cancer) to the CNS presents a significant clinical challenge, often leading to poor prognosis and early death. BriaCell has recently conducted a retrospective analysis in breast cancer patients with CNS metastases enrolled across both its Bria-IMT™ monotherapy and combination therapy with CPI(immune checkpoint inhibitors) studies and found remarkable clinical responses	CNS-ORR:71%

ID, identifier; BMs: brain metastasis; BCBM: breast cancer with brain metastasis; BC: breast cancer; CR: complete response; CNS-ORR: central nervous system objective response rate; MBC: metastasis breast cancer; mPFS: median progression-free survival; mo: months; NC: no change; ORR: objective response rate; PR: partial response; T-DX: dtrastuzumab deruxtecan

### Chemotherapy

#### Cisplatin

Previous findings have highlighted the significant activity of cisplatin (P) combined with etoposide (E) in BCBM [70]. A prospective study aimed to evaluate the first-line efficacy of this combination in BCBM patients who had not undergone prior radiotherapy. The results demonstrated promising outcomes, with 7 out of 56 patients achieving complete response (CR) (13%) and 14 achieving partial response (PR) (25%), yielding an ORR of 38%. These findings confirm the effectiveness of platinum-based combination therapy for BCBM patients [71]. In another study assessing the efficacy and tolerability of cisplatin combined with gemcitabine in BCBM, subgroup analysis revealed a markedly higher ORR of 66.6% for TNBC patients compared to 25% for HR + patients. The mPFS for TNBC patients was 5.6 months (95% CI 2.4–8.8 months), surpassing the 3.6 months observed in HR + patients. Notably, TNBC patients receiving this regimen as first-line treatment achieved a mPFS of 9.2 months (95% CI 5.2–13.2 months) [72]. These results underscore the efficacy of platinum-based chemotherapy regimens, particularly in the TNBC subtype of BCBM. Nevertheless, further prospective and randomized trials are essential to establish more definitive and conclusive evidence.

#### Capecitabine

Capecitabine, an oral antimetabolite drug, and its metabolites are capable of crossing the BBB, making it a viable option for maintenance therapy following radiotherapy or local treatments [73]. Gouveia et al. conducted a retrospective analysis of 209 BCBM patients treated with capecitabine monotherapy post-surgery or radiotherapy, with TNBC cases comprising 26.4% of the cohort. After a median follow-up of 6 months, the results demonstrated a higher CNS-ORR for TNBCBM compared to other molecular subtypes. However, the 3-month disease control rate for TNBCBM was lower, with a mPFS of 3.0 months and a mOS of 4.5 months [74]. Moreover, studies have shown that combining capecitabine with radiotherapy can enhance local control rates in TNBCBM. However, this combination did not yield significant improvements in OS compared to radiotherapy alone [75]. In summary, while capecitabine is a feasible option for maintenance therapy in TNBCBM, its clinical efficacy remains limited.

#### Utidelone

Utidelone, a novel epothilone-class microtubule inhibitor, is a non-P-glycoprotein substrate, enabling it to bypass the multidrug resistance mechanism mediated by P-glycoprotein on cancer cell membranes. This property allows utidelone to effectively cross the BBB and sustain high concentrations in brain tissue, as confirmed in preclinical brain tissue distribution studies and cerebrospinal fluid tests in patients, highlighting its potential for treating BCBM [76]. Recent clinical findings further underscore its promising efficacy. At the 2023 ESMO conference, preliminary data from the UTOBIA-BM study involving 17 patients demonstrated a CNS-ORR of 73% and a CNS clinical benefit rate (CNS-CBR) of 91%, reflecting significant anti-tumor activity, the study is ongoing [77]. At the recent ASCO conference, a Phase II clinical trial (U-BOMB) evaluating utidelone in combination with bevacizumab for HER2- BCBM included 47 patients—35 with newly diagnosed active BM (without prior local treatment) and 12 with progressive disease following brain radiotherapy. The CNS-ORR for the overall cohort was 42.6%. With a median follow-up of 14.6 months (as of January 8, 2024), the mPFS was 7.7 months (95% CI 5.5–10.8). Subgroup analysis revealed a CNS-ORR of 33.3% and a mPFS of 5.9 months for HR + /HER2- patients, while TNBC patients achieved a CNS-ORR of 55% with a mPFS of 8.4 months. The safety profile of utidelone was favorable, with adverse events primarily limited to grade 1–2 severity [78]. Based on these promising data, the FDA granted utidelone orphan drug designation for the treatment of BCBM in March 2024.

#### Temozolomide

TMZ, an alkylating agent with excellent CNS penetration, is FDA-approved for glioblastoma treatment. Preclinical evidence, including mouse models, has demonstrated the potential of low-dose, preventive, rhythmic TMZ in reducing BM.A clinical study evaluated TMZ combined with vincristine and radiotherapy in BCBM patients without prior BM treatment. The study included 36 evaluable patients, 2 of whom had HER2 overexpression. The results were promising, with 3 patients achieving CR and 16 achieving PR, leading to an ORR of 52% (95% CI 38–67%), surpassing the predefined efficacy threshold. The mPFS was 8 months, while the mOS reached 11 months. The regimen exhibited favorable tolerability, with predominantly mild side effects. Furthermore, analysis of functional status using the FACT-B (Functional Assessment of Cancer Therapy-Breast) tool revealed significant improvements in patient quality of life during the study period. This underscores the dual benefit of the treatment in achieving favorable clinical outcomes and enhancing patient well-being [79].Given the observed efficacy of TMZ in combination with the ADC drug T-DM1 for HER2 + BCBM, further investigation into TMZ-based combination regimens, particularly in TNBCBM patients, holds significant potential for advancing treatment strategies.

### Targeted therapy

#### PARP inhibitors

Poly(ADP-ribose) polymerase (PARP) inhibitors are a novel class of targeted therapies that exploit synthetic lethality to selectively target tumors with DNA damage repair defects. They have revolutionized the treatment of germline BRCA1/2 (gBRCA1/2)-associated breast cancer, marking a significant advancement in hereditary tumor management [80]. BRCA1/2 mutations occur in approximately 5 to 10% of breast cancer cases and are more prevalent in patients with a family history of breast cancer, younger age at diagnosis, and TNBC [81]. Preclinical studies have demonstrated that BRCA1/2-mutant tumor cells are highly sensitive to PARP inhibitors due to defects in homologous recombination repair (HRR). When combined with DNA-damaging chemotherapies, PARP inhibitors prevent effective DNA repair, leading to the accumulation of DNA damage and apoptosis in tumor cell. In MBC, nearly half of patients with BRCA mutations eventually develop BM, and PARP inhibitors provide a promising targeted therapeutic option for these patients [82].

Despite their potential, clinical development of PARP inhibitors has faced challenges, particularly due to hematologic toxicity in combination with chemotherapy. Veliparib, a first-generation PARP inhibitor, stands out for its antitumor activity and manageable safety profile. It can be administered as monotherapy or combined with platinum-based chemotherapy for metastatic TNBC and BRCA1/2-associated breast cancer. Although the study included a subgroup of patients with progressive BM (n = 9), specific outcomes for this group remain unreported [83].

The phase III OlympiA trial established olaparib as a superior option compared to chemotherapy in HER2-negative MBC patients with germline BRCA mutations. Olaparib demonstrated a significant improvement in PFS (PFS: 7.0 months vs. 4.2 months; HR = 0.58, P < 0.001) with a favorable safety profile, leading to its accelerated FDA approval [84]. Subgroup analysis showed substantial PFS benefit in the TNBC group (HR = 0.47; 95% CI 0.32–0.69). While specific data on BM were not analyzed [85], case studies have reported olaparib's efficacy in controlling BM in BRCA-mutant patients [86].

Talazoparib, a more potent PARP inhibitor, operates via dual mechanisms: PARP enzyme inhibition and PARP trapping at DNA damage sites. This dual action enhances its effectiveness in BRCA-mutated cancers [87]. Approved by the FDA in 2018 for HER2-negative locally advanced or MBC with pathogenic or suspected gBRCA mutations, talazoparib has shown exceptional clinical performance. The phase III EMBRACA trial demonstrated significantly improved PFS with talazoparib compared to chemotherapy (mPFS: 8.6 months vs. 5.6 months; P < 0.0001), reducing the risk of progression by 46% and doubling the ORR (ORR: 62.6% vs. 27.2%; P < 0.0001). Notably, the PFS benefit was consistent across all predefined subgroups, including patients with a history of BM [88].

These findings position PARP inhibitors, particularly olaparib and talazoparib, as highly promising targeted therapies for patients with BRCA-mutant breast cancer, including those with BM. Further research is warranted to fully elucidate their efficacy in CNS disease control and explore synergistic combinations for enhanced therapeutic outcomes.

#### Anti-angiogenic therapy

Bevacizumab (BEV), a humanized monoclonal antibody targeting vascular endothelial growth factor (VEGF), exerts its anti-angiogenic effect by blocking VEGF binding to its receptors, thereby inhibiting the formation of new blood vessels. Although the use of BEV in breast cancer remains a topic of debate, it has demonstrated notable benefits in terms of PFS and ORR, particularly in TNBC patients. Recent studies have also highlighted its potential role in the treatment of BCBM. Firstly, BEV has been shown to enhance the efficacy of chemotherapy, improving both CNS-ORR and PFS. Research indicates that combining BEV with eribulin and etoposide significantly increases CNS-ORR in patients withBM [77]; Additionally, the combination of BEV with eribulin has demonstrated a substantial improvement in CNS-ORR and PFS specifically in TNBC patients [78]. Secondly, BEV may potentiate the effects of radiation therapy. The phase II A-PLUS trial evaluated the combination of BEV, etoposide, and cisplatin (BEEP) as induction therapy prior to whole-brain radiation therapy (WBRT). Results showed an improvement in CNS-PFS, with a mPFS of 8.1 months compared to 6.5 months (HR = 0.71; 95% CI 0.44–1.13; P = 0.15), meeting significance at the pre-specified α = 2.0 level. At 8 months, the CNS-PFS rate was significantly higher in the BEEP group compared to the control group (48.7 vs. 26.3%; P = 0.03) [89]. Thirdly, BEV has demonstrated the ability to reduce the risk of BM. Preclinical studies in breast cancer animal models have shown that combining VEGF and angiopoietin-2 inhibition can lower the incidence of BM, underscoring the preventive potential of anti-angiogenic strategies [90]. In summary, while real-world evidence supports the promising role of BEV in treating BCBM, particularly in combination therapies, the lack of high-quality prospective trials remains a limitation. Further rigorous studies are essential to validate the efficacy and establish the clinical utility of BEV-based regimens for BCBM patients.

### ADCs

#### Sacituzumab Govitecan (SG)

Based on the results of the ASCENT trial, sacituzumab govitecan (SG) has been approved for patients with treatment-resistant TNBC. Among the trial participants, 61 patients had stable BM. The findings demonstrated that SG provided significant clinical benefits in PFS and OS, regardless of BM presence, when compared to physician-selected chemotherapy. Specifically, SG extended PFS to 5.6 months versus 1.7 months (HR = 0.41; 95% CI 0.32–0.52; P < 0.001) and OS by 5.4 months (12.1 months vs. 6.7 months; HR = 0.48; 95% CI 0.38–0.59; P < 0.001) [91]. While BM-specific outcomes were not directly analyzed, the results highlight SG’s robust efficacy, warranting future exploration of SG-based combination therapies for TNBC patients with BM. Notably, the domestically developed Trop-2-targeted ADC SHR-A1921 is under evaluation in a phase II trial (NCT06210438), investigating its combination with bevacizumab for TNBC patients with BM.

#### T-DXd

T-DXd has demonstrated exceptional efficacy across HER2 + BC subtypes, including treatment-naive and pre-treated cases, patients with stable and active BM, as well as those with leptomeningeal metastasis. The DESTINY-Breast 04 trial marked a breakthrough as the first phase III study to target HER2-low expressing MBC, confirming significant benefits in PFS and OS [92]. Among the 63 HER2-low expressing TNBC patients (11.3% of the cohort), a smaller subgroup that reflects the prevalence of HER2-low expression in the TNBC population, T-DXd significantly outperformed chemotherapy, with a mPFS of 8.5 months versus 2.9 months (95% CI 4.3–11.7; HR = 0.46) and a mOS of 18.2 months versus 8.3 months (95% CI 13.6–NE; HR = 0.48). The ORR for T-DXd was 50.0%, nearly triple that of chemotherapy (16.7%). Although outcomes for the seven patients with BM were not analyzed separately, these findings strongly support further investigation of T-DXd in TNBCBM.

T-DXd has already demonstrated significant intracranial efficacy in HER2 + BC patients with BM [66]. However, its role in HER2-low expressing patients with BM remains under evaluation. According to the RANO-BM criteria, the DEBBRAH study provided encouraging evidence in HER2-low expressing, heavily pre-treated MBC with BM patients. The study’s second cohort (n = 6, untreated asymptomatic BM) and fourth cohort (n = 6, BM progressing after local treatment) reported promising intracranial responses. In the untreated cohort, the iORR was 50.0% (3/6 patients; 95% CI 11.8–88.2%), while the post-local treatment cohort achieved an ORR of 33.3% (2/6 patients; 95% CI 4.3–77.7%; P = 0.033, one-sided). All responders achieved PR, with a median intracranial response duration of 7.2 months and a median time to intracranial response of 2.3 months [93]. In conclusion, while the findings are promising, larger prospective trials are needed to validate the efficacy of T-DXd in HER2-low expressing MBC patients with BM and further define its role in this challenging population.

### Immunotherapy

Immunotherapy has rapidly evolved into a standard treatment for multiple cancer types, including TNBC.CPIs, such as the anti-PD-L1 antibody atezolizumab and the anti-PD-1 antibody pembrolizumab, have been approved for use in combination with chemotherapy as first-line therapy for PD-L1-positive (PD-L1 +) advanced TNBC. This approval stems from the significant benefits observed in PFS, ORR, and OS in the IMpassion130 and KEYNOTE-355 trials, where CPI-chemotherapy combinations demonstrated clear superiority over chemotherapy alone [94, 95].

Traditionally, the brain was regarded as an *"immune-privileged site"* due to its specialized anatomical and physiological barriers. However, recent research has identified functional lymphatic vessels in the meninges, which facilitate lymphocyte drainage and immune cell trafficking. Furthermore, BM can compromise BBB, allowing immune cells and therapeutic agents to infiltrate the brain microenvironment. While immunotherapy has shown remarkable success in BM from melanoma and non-small cell lung cancer (NSCLC)—as evidenced in studies such as those by Long et al. [96] and the KEYNOTE-189 trial [97]. Experience with intrathecal administration of trastuzumab in breast cancer and rituximab in lymphoma patients has demonstrated that intrathecal injection significantly increases drug exposure in CSF and provides clinical benefits. A study published in *Nature Medicine* in 2022 on intrathecal administration of nivolumab, although focused on melanoma-associated LMD, offers valuable insights for breast cancer and lung cancer LMD as well[98]. This study represents an important step forward, providing a new option for patients with LMD from solid tumors. Data on CPIs in BCBM remains limited, nevertheless, these findings underscore the potential of immunotherapy for BCBM, warranting further investigation.

Beyond CPIs, other immunotherapy approaches are actively being explored in TNBC. Chimeric antigen receptor T (CAR-T) cell therapy has demonstrated strong specificity and antitumor activity in EGFR-high expressing TNBC [99]. Additionally, Bria-IMT^™^, an emerging cellular immunotherapy, has shown notable efficacy in patients with advanced BCBM. In a study involving seven patients, five achieved significant intracranial tumor reduction, yielding an iORR of 71%. Notably, Bria-IMT^™^ provided substantial clinical benefit even in heavily pretreated MBC patients, including those who had previously failed ADC therapy [100].These findings highlight the growing potential of immunotherapy, including CPI-based regimens, CAR-T cell therapies, and novel immunotherapeutic approaches such as Bria-IMT™, in addressing the therapeutic challenges posed by TNBC and its associated BM. Further large-scale clinical studies are crucial to validate these promising results and optimize immunotherapy strategies for BCBM.

### Discussion

Overall, treatment strategies for BM in TNBC are advancing toward precision and individualized approaches. While traditional chemotherapy remains the cornerstone of management, novel chemotherapeutic agents, targeted therapies, ADCs, and immunotherapy have demonstrated promising efficacy, offering renewed hope to patients with TNBCBM. Nevertheless, the pursuit of high-quality prospective randomized trials and enhanced interdisciplinary collaboration remains pivotal for future advancements in this field.

## Dreams of the future

Given the distinct molecular profiles of BM compared to primary tumors and other metastatic sites, a comprehensive understanding of the genomic landscape and tumor microenvironment dynamics within intracranial lesions is essential for implementing precision treatments. Future directions for managing BCBM include targeted therapies aimed at the BM microenvironment, strategies to enhance BBB permeability, optimization of existing targeted therapies, immunotherapy, multimodal treatment combinations, and the integration of CSF circulating tumor DNA (ctDNA) for real-time monitoring of tumor dynamics. Moreover, identifying and validating novel biomarkers to pinpoint patients more likely to respond to specific therapies is imperative.

While most treatment approaches for BCBM remain in the clinical trial phase, emerging therapies—such as T-DXd, GRN1005, BEV, and immunotherapy, along with interventions targeting the BM microenvironment—have demonstrated significant potential. With the completion of ongoing clinical trials and deeper insights into the mechanisms driving BM, these innovative treatments are anticipated to play a transformative role in the management of BCBM. Such progress holds promise for improving survival outcomes and bringing renewed hope to affected patients in the near future.

## Data Availability

No datasets were generated or analysed during the current study.
